# Editorial: Exploring the key targets and compounds that manipulate brain neurocircuits against mental disorders and psychiatric volume II

**DOI:** 10.3389/fphar.2025.1601881

**Published:** 2025-04-14

**Authors:** Weijie Xie, Yuan Li, Song Zhang

**Affiliations:** ^1^ Shanghai Pudong New Area Mental Health Center, Tongji University School of Medicine, Shanghai, China; ^2^ Shanghai Key Laboratory of Psychotic Disorders, Brain Health Institute, National Center for Mental Disorders, Shanghai Mental Health Center, Shanghai Jiaotong University School of Medicine, Shanghai, China; ^3^ Department of Anesthesiology, Renji Hospital, School of Medicine, Shanghai Jiao Tong University, Shanghai, China

**Keywords:** neurophamacology, neurocircuit, brain network, molecular target, compoud

## Introduction

More than 970 million people worldwide suffer from mental disorders, which remain a major cause of disability despite advancements in pharmacological treatments (Chen et al., 2025; Syed et al., 2025). Understanding and treating mental disorders involves comprehending the complex interactions between molecular mechanisms, neural circuits, and behavioral outcomes. This Research Topic “*Exploring the Key Targets and Compounds that Manipulate Brain Neurocircuits Against Mental Disorders and Psychiatric Diseases Volume II*”, compiles 12 innovative studies that collectively advance our knowledge of neurocircuit modulation through diverse pharmacological, genetic, and systems-level approaches. These contributions highlight emerging therapeutic strategies, mechanistic insights, and technological advancements, offering a roadmap for future breakthroughs in psychiatry and neuropharmacology.

## Targeting neuroinflammation and oxidative stress

Neuroinflammation and oxidative stress are pivotal contributors to neurodegenerative and psychiatric disorders. Two studies exemplify how novel compounds can mitigate these processes. Hou et al. synthesized 2H-1,4-benzoxazin-3(4H)-one derivatives fused with 1,2,3-triazole, demonstrating potent anti-inflammatory effects in LPS-stimulated microglia. These compounds suppressed pro-inflammatory cytokines (IL-1β, IL-6, TNF-α) and activated the Nrf2-HO-1 pathway, underscoring their dual antioxidant and anti-inflammatory potential. Similarly, Lai et al. revealed that (+)-catechin alleviates corticosterone-induced oxidative stress and pyroptosis in PC12 cells by activating PI3K/AKT and Nrf2/HO-1/NF-κB pathways. Both studies emphasize the therapeutic promise of targeting oxidative-inflammatory cascades, particularly through Nrf2 activation, to preserve neuronal integrity.

## Advancing neuroimaging and neurogenesis research

Cutting-edge neuroimaging techniques continue to unravel how neuromodulators and disease states reshape brain networks. Hagan et al. demonstrated that intranasal oxytocin enhances small-world topology in resting-state networks, particularly in regions governing social cognition. This suggests oxytocin’s therapeutic potential lies in optimizing information flow within critical circuits. Complementing this, Liu et al. employed bibliometric analysis to identify adult hippocampal neurogenesis (AHN) as a hotspot, linking its dysfunction to Alzheimer’s disease and anxiety. Meanwhile, Che et al. used ^1^H-MRS to correlate hippocampal metabolite ratios with cognitive decline in general paresis patients, highlighting neuroimaging’s role in tracking neurodegeneration. Together, these studies illustrate how multimodal imaging and meta-analyses can bridge molecular changes to brain neurocircuit-level dysfunctions.

## Pharmacological interventions: efficacy and metabolic challenges

The efficacy and side effects of psychotropic drugs remain central to clinical psychiatry. Zhao et al. compared five antidepressants in cancer patients, revealing escitalopram, duloxetine, and vortioxetine as superior to sertraline, with trazodone augmentation enhancing outcomes. However, metabolic side effects of antipsychotics, such as olanzapine-induced weight gain, pose significant challenges. Huang et al. identified lipid metabolism dysregulation in the lateral septum as a key driver of olanzapine’s adverse effects, implicating APOA1/APOC3/APOH genes. Conversely, Zhang et al. demonstrated that the α_2_-adrenoceptor agonist dexmedetomidine alleviates pain by restoring neuronal metabolism and spinal perfusion, suggesting metabolic modulation as a dual therapeutic strategy. These findings underscore the need for precision medicine to balance efficacy and safety.

## Unraveling genetic and molecular mechanisms

Genetic variants and molecular pathways underlying neurodevelopmental and neurodegenerative disorders are increasingly elucidated. Xue et al. linked a novel FLNA frameshift variant to periventricular nodular heterotopia, showing disrupted F-actin organization in patient-derived iPSCs. Similarly, Guo et al. implicated EphrinB2/EphB2 signaling in maternal separation-induced visceral hyperalgesia, where spinal glia-neuron crosstalk drives pain sensitization. Multi-omics approaches further illuminated peripheral biomarkers in multiple sclerosis, with B-cell expansion and reduced sphingolipids distinguishing subtypes (Zhou et al.). These studies demonstrate how genetic, cellular, and omics tools can unravel the mechanisms of disease and pinpoint potential therapeutic targets.

## Neuroprotection and mitochondrial dynamics

Mitochondrial dysfunction is a common thread in brain injury and neurodegeneration. Zhu et al. demonstrated that Dl-3-n-butylphthalide (NBP) mitigates cerebral ischemia/reperfusion injury by enhancing mitochondrial fusion via AMPK/Mfn1 activation. This aligns with broader efforts to harness mitochondrial dynamics as a neuroprotective strategy, providing hope for stroke and related disorders.

## Conclusion and future prospects

This Research Topic underscores the complexity of mental disorders and the need for interdisciplinary approaches, including multi-target therapies (e.g., Nrf2 activators, PI3K/AKT enhancers), precision neuroimaging, genetic and omics-driven insights, and mitigating drug side effects. Future research should prioritize translational studies bridging preclinical findings to clinical trials, leveraging emerging technologies like single-cell omics and AI-driven drug discovery. By integrating these avenues, we advance our understanding and ability to manipulate the neural circuits related to mental health, ultimately aiming to apply these advancements in clinical settings.
